# Sputum lipoarabinomannan (LAM) as a biomarker to determine sputum mycobacterial load: exploratory and model-based analyses of integrated data from four cohorts

**DOI:** 10.1186/s12879-022-07308-3

**Published:** 2022-04-02

**Authors:** Aksana Jones, Jay Saini, Belinda Kriel, Laura E. Via, Yin Cai, Devon Allies, Debra Hanna, David Hermann, Andre G. Loxton, Gerhard Walzl, Andreas H. Diacon, Klaus Romero, Ryo Higashiyama, Yongge Liu, Alexander Berg

**Affiliations:** 1 Simulations Plus, Inc, 42505 10th Street W, Lancaster, CA 93534 USA; 2 DST-NRF Centre of Excellence for Biomedical Tuberculosis Research; South African Medical Research Council Centre for Tuberculosis Research; Division of Molecular Biology and Human Genetics, Faculty of Medicine and Health Sciences, Stellenbosch University, Cape Town, South Africa; 3 Tuberculosis Research Section, Laboratory of Clinical Microbiology and Immunology, Division of Intramural Research, National Institute of Allergy and Infectious Disease, National Institutes of Health, Bethesda, MD USA; 4 Institute of Infectious Disease and Molecular Medicine, Faculty of Health Science, University of Cape Town, Cape Town, South Africa; 5 Bill & Melinda Gates Foundation, Seattle, Washington, USA; 6 TASK Applied Science, Cape Town, South Africa; 7 Critical Path Institute, Tucson, AZ USA; 8 Otsuka Pharmaceutical Company, Tokushima, Japan; 9 Otsuka Pharmaceutical Development & Commercialization, Inc, Rockville, MD USA

**Keywords:** Tuberculosis, Lipoarabinomannan, LAM, Biomarker, *Mycobacterium*

## Abstract

**Background:**

Despite the high global disease burden of tuberculosis (TB), the disease caused by *Mycobacterium tuberculosis* (*Mtb*) infection, novel treatments remain an urgent medical need. Development efforts continue to be hampered by the reliance on culture-based methods, which often take weeks to obtain due to the slow growth rate of *Mtb*. The availability of a “real-time” measure of treatment efficacy could accelerate TB drug development. Sputum lipoarabinomannan (LAM; an *Mtb* cell wall glycolipid) has promise as a pharmacodynamic biomarker of mycobacterial sputum load.

**Methods:**

The present analysis evaluates LAM as a surrogate for *Mtb* burden in the sputum samples from 4 cohorts of a total of 776 participants. These include those from 2 cohorts of 558 non-TB and TB participants prior to the initiation of treatment (558 sputum samples), 1 cohort of 178 TB patients under a 14-day bactericidal activity trial with various mono- or multi-TB drug therapies, and 1 cohort of 40 TB patients with data from the first 56-day treatment of a standard 4-drug regimen.

**Results:**

Regression analysis demonstrated that LAM was a predictor of colony-forming unit (CFU)/mL values obtained from the 14-day treatment cohort, with well-estimated model parameters (relative standard error ≤ 22.2%). Moreover, no changes in the relationship between LAM and CFU/mL were observed across the different treatments, suggesting that sputum LAM can be used to reasonably estimate the CFU/mL in the presence of treatment. The integrated analysis showed that sputum LAM also appears to be as good a predictor of time to Mycobacteria Growth Incubator Tube (MGIT) positivity as CFU/mL. As a binary readout, sputum LAM positivity is a strong predictor of solid media or MGIT culture positivity with an area-under-the-curve value of 0.979 and 0.976, respectively, from receiver-operator curve analysis.

**Conclusions:**

Our results indicate that sputum LAM performs as a pharmacodynamic biomarker for rapid measurement of *Mtb* burden in sputum, and thereby may enable more efficient early phase clinical trial designs (e.g., adaptive designs) to compare candidate anti-TB regimens and streamline dose selection for use in pivotal trials.

*Trial registration* NexGen EBA study (NCT02371681)

**Supplementary Information:**

The online version contains supplementary material available at 10.1186/s12879-022-07308-3.

## Background

Although temporarily eclipsed by the COVID-19 pandemic, tuberculosis (TB), the disease caused by *Mycobacterium tuberculosis* (*Mtb*), remains the largest infectious disease-related cause of death worldwide [1]. The significant mortality associated with TB and the slow rate of progress in decreasing the global burden of the disease highlight the need for improved treatments. Towards this end, the identification of novel treatment regimens that are highly efficacious against susceptible and drug-resistant strains of *Mtb* as well as treatment regimens that are significantly shorter in duration than the current 6-month standard-of-care regimen for drug-susceptible pulmonary TB has become a focus of current development efforts [2].

However, many hurdles are handicapping the development of such treatment shortening regimens [3]. One is the lack of real-time biomarkers to rapidly gauge treatment response in TB drug development clinical trials [4]. Results from sputum culture-based methods are the only accepted early marker of efficacy and sustained sputum negativity is the backbone of the definition of cure [5, 6]. Due to the inherently slow growth rate of *Mtb*, the reliance on culture-based methods results in a significant lag between sample collection and availability of results, potentially up to 6 weeks or 2 months in the case of liquid and solid media culture, respectively. This presents a problem in everyday practice in that a patient can be lost to follow-up before their results are known. In the clinical trial setting, a real-time biomarker would enable researchers to utilize adaptive clinical study designs that have been demonstrated to improve clinical development efficiency and knowledge generation in other therapeutic areas [7, 8].

Previously, it has been shown that sputum lipoarabinomannan (LAM) concentrations, as measured by an immunoassay developed by Otsuka Pharmaceutical Co., Ltd., (hereafter called LAM-ELISA) has the potential to be such a “real-time” pharmacodynamic (PD) biomarker [9]. However, the relationship between sputum LAM and bacterial burden was initially only studied using time-to-detection (TTD) from Mycobacteria Growth Incubator Tube (MGIT)-culture, and during standard isoniazid, rifampin, pyrazinamide, and ethambutol treatment in 40 patients. The quantitative nature of the assay had not been rigorously evaluated in patients during treatment using colony-forming units (CFU) from solid medium, the gold-standard of quantifying bacterial load. Furthermore, it is not known whether the relationship between sputum LAM and quantitative culture results is impacted by treatment with TB drugs that have different mechanisms. To address these questions, we measured LAM concentrations from sputa obtained from participants of the “NexGen EBA” [10] trial (NCT02371681) and compared them with CFU and MGIT assay-based time to detection (MGIT-TTD) obtained from the same sputum specimen. Further, we performed an integrated data analysis by combining NexGen EBA data with data from 3 additional cohorts: 1 cohort from biobanked sputum specimens from TB-treatment–naive participants and 2 cohorts from a previously published study that included both TB-treatment–naive participants and TB patients undergoing standard-of-care treatment for the initial 56 days [9]. The integrated analysis compared sputum positivity using LAM versus using culture results. The exploratory and model-based analyses presented herein are intended to serve as a basis for understanding the benefits and limitations of sputum LAM to further guide development and inform biomarker qualification efforts.

## Methods

### Study design and data

Data obtained from 4 cohorts were utilized for the current analysis, as summarized in Table 1. This included the NexGen EBA cohort that examined the sputum LAM concentration during 14-day TB treatment [10] and a biobanked sputum specimen cohort that examined sputum culture and LAM in TB-treatment-naive participants. Additionally, data from 2 cohorts previously published were included: the first evaluated sputum LAM concentrations in treated TB patients and the other in treatment-naive patients [9]. Informed consent was obtained from all participants. In the integrated analysis, participant-level data was pooled, such that data from each individual participant was combined into a single aggregated dataset across all 4 cohorts while retaining participant-specific information.

**Table 1:** Overview of included cohorts, number of participants and number of observations

Cohort Name	NexGen EBA Cohort^a^	Biobank Sputum Specimen Cohort	Kawasaki Study Cohorts^b^	
Cohort Type	Treatment Clinical study NCT02371681^a^	Diagnostic In vitro study using biobanked sputum samples	Diagnostic	Treatment
Participants (n)	178	250	308	40
Cohort Population	Adults with smear (+) pulmonary TB undergoing treatment for drug-susceptible TB	Adults suspected of pulmonary TB prior to treatment, and adult participants without TB	Adults suspected of pulmonary TB prior to treatment, and adult participants without TB	Adults with smear (+) pulmonary TB undergoing treatment for drug-susceptible TB
Treatment Regimen(s) (n)	Arm 1: Isoniazid (H) (22) Arm 2: Rifampin (R) (22) Arm 3: Pyrazinamide (Z) (22) Arm 4: Moxifloxacin (M) (23) Arm 5: R + Z (23) Arm 6: H + Z (22) Arm 7: MRZE (22) Arm 8: HRZE (22)	Diagnostic (250)	Diagnostic (308)	Standard of care regimen: Isoniazid (H), Rifampin (R), Pyrazinamide (Z), and Ethambutol (E): HRZE (40)
Duration (days)	14	Pre-treatment only	Pre-treatment only	56
Pharmacodynamic Response Measures (N)	1. AFB smear grade (Screening only) 2. CFU concentration (2323) 3. LAM concentration direct (439) and sediment (445) 4. MGIT-TTD (2459) 5. Xpert Cycle Threshold (Screening only)	1. AFB smear grade 2. Culture positivity (LJ media and MGIT [binary status]) 3. LAM concentration direct (250)	1. AFB smear grade 2. Culture positivity (LJ media and MGIT [binary status]) 3. LAM concentration direct (308) and sediment (308) 4. MGIT-TTD (271) 5. Xpert Cycle Threshold	1. AFB smear grade 2. LAM concentration direct (202) and sediment (175) 3. MGIT-TTD (180)
Sampling Days^c^	Screening, Baseline, Day 7, and Day 13	Baseline (pre-treatment)	Baseline (pre-treatment)	Baseline, Day 3, Day 7, Day 14, Day 28, and Day 56

AFB, acid-fast bacilli; CFU, colony-forming units; EBA, early bactericidal activity; FIND, Foundation for Innovative New Diagnostics; LAM, lipoarabinomannan; LJ, Löwenstein-Jensen; N, number of records; n, number of participants; MGIT, Mycobacterium Growth Indicator Tube; TTD, time to detection

^a^NexGen EBA Radiologic and Immunologic Biomarkers of Sterilizing Drug Activity in Tuberculosis. ClinicalTrials.gov (2017). Available at: https://clinicaltrials.gov/ct2/show/NCT02371681. (Accessed: 02 August 2017)

^b^Kawasaki M, Echiverri C, Raymond L, Cadena E, Reside E, Tarcela Gler M, et al. Lipoarabinomannan in sputum to detect bacterial load and treatment response in patients with pulmonary tuberculosis: analytic validation and evaluation in two cohorts. PLoS Med. 2019;16(4):e1002780

^c^Additional sampling timepoints were collected for CFU and MGIT-TTD in NexGen EBA Study: daily from Day 1 through Day 14

#### NexGen EBA cohort

For details of the study, please see NCT02371681 and Xie, et al. [10]. Sputum specimens were collected for 16 h overnight daily during a 14-day treatment. Sputum for CFU counts of *Mtb* and measurement of time to positivity in liquid culture medium (BACTEC MGIT 960, Becton Dickinson) was subject to laboratory processing centrally at the TASK laboratory (TASK Applied Science, Cape Town, South Africa), performed according to standard operating procedures established at TASK [11]. While quantitative culture was performed for all sputum specimens, LAM was measured for the Baseline, Day 7, and Day 13 samples. Therefore, only data from Baseline, Day 7, and Day 13 were analyzed.

#### Biobank sputum specimen cohort

Foundation for Innovative New Diagnostics (FIND; Geneva, Switzerland) provided 250 banked sputum specimens from 250 participants (each participant provided 1 sputum specimen), obtained in Vietnam under FIND’s “Generic Protocol: TB Reference Materials-Collection, Storage & Distribution.” During the sample collection, 2 sputum specimens, an early morning sputum and a spot sputum, were collected from participants who were presented to the clinic with symptomatic pulmonary disease thought to be TB. Enrollment depended upon routine passive case detection. Sputum specimens were examined with concentrated Ziehl Neelsen AFB (acid-fast bacilli) staining smear microscopy, and by Löwenstein–Jensen (LJ) and MGIT culture of NALC-NaOH decontaminated sediments. The final diagnosis was based on the clinical and laboratory data of the 2 sputum specimens as (1) TB, smear and positive; (2) TB, smear negative but culture positive; and (3) non-TB (no evidence of TB from chest X-ray, smear microscopy, or culture). FIND selected 1 sputum from each participant of the banked 2 samples and provided for LAM measurements. There were 85 early morning sputa and 165 spot sputa.

#### Data from a previously published study

For details, please see Kawasaki, et al. [9]. The included data were from 2 cohorts: 1 included non-TB (no evidence of TB from chest X-ray, smear microscopy, or culture) and TB-treatment–naive participants and the other included TB patients undergoing standard-of-care treatment for the initial 56 days.

### Measurements of efficacy/biomarkers

The following outcome variables reflecting bacterial burden were utilized in the analyses:

- *LAM concentration: *LAM was measured using LAM-ELISA [9], and was a continuous variable with a lower limit of quantitation (LLOQ) of 15 pg/mL. LAM was measured on direct sputum samples, that is, the same sample split for solid medium culture, and on processed sputum samples, that is, the sediment used for MGIT inoculation, except for the biobanked specimen cohort where LAM was only obtained from the direct sputum samples since MGIT culture was not done for those samples.
- *CFU concentration:* CFU counts per mL were obtained only in the NexGen EBA cohort as a continuous variable.
- *MGIT-TTD:* a continuous variable indicating time to positive signal in the MGIT assay, with an upper limit of quantitation (ULOQ) of 42 days (1008 h).
- *LAM positivity:* a binary variable ( ±) indicating whether the sputum LAM concentration was above or below the LLOQ of 15 pg/mL.
- *Solid culture positivity:* a binary variable ( ±) indicating whether mycobacterial growth was observed via solid media culture of sputum samples (7H11 or LJ media used in NexGen EBA cohort, and the Kawasaki diagnostic sample cohorts and biobank sample cohort, respectively). The quantitative culture using 7H11 media has a lower limit of detection (LLOD) of 20 CFU/mL (this was calculated with the minimum of 1 CFU countable from 50 μL of sputum spread on each plate). Solid media culture used in routine diagnostics, such as LJ, has an estimated LLOD of approximately 100 CFU/mL [12].
- *MGIT assay-based culture positivity:* a binary variable ( ±) indicating whether mycobacterial growth was observed via liquid media culture of sputum samples, assuming negativity where the MGIT-TTD exceeds the 42-day ULOQ.

In binary assessment of sample positivity/negativity, data points for LAM that were < LLOQ and for MGIT-TTD that were > ULOQ were declared as “negative” samples. These samples were treated as missing in the rest of the analysis. For the pairwise comparisons between variables, data points were excluded if either of the compared variables was missing. Any observations with missing timepoint identifications were excluded.

#### Correlation between assays

Correlations between assays were calculated using the data pooled across all 4 cohorts. All comparisons were made using data from paired samples collected from the same participant (that is, within-participant comparison). Paired samples were defined as time-matched samples collected within the same individual at the same study visit (for example, Baseline).

Previous analyses indicated that both LAM (data unpublished) and CFU [13] concentrations are log-normally distributed and, thus, are presented in the current analysis as base 10 logarithm (log_10_)-transformed values, whereas relationships between LAM and MGIT-TTD are presented using both log_10_-transformed values and linear scale values.

Longitudinal trends were assessed by calculating change from Baseline (CFB) values for CFU and LAM concentrations or for MGIT-TTD, where CFB values at the Baseline visit (Day 0) were assigned a value of zero.

### Model-based analyses

#### Assessment of treatment regimens during model development

In the model-based analyses described subsequently, the impact of treatment regimens was of specific interest. Thus, exploratory data analysis was performed, whereby tabular and graphical summaries were created with stratification by treatment regimen to evaluate whether trends were present. Additionally, to determine whether a significant treatment effect was apparent, each treatment regimen was added as separate fixed-effect (covariate) in the analyses. These fixed-effects were evaluated for statistical significance and explored for possible simplifications of treatment groups that could be redefined using fewer groups, which were then iteratively tested.

#### Model-based analysis of the relationship between sputum LAM concentration and solid media-based CFU count

A power model approach was used to determine the relationship between sputum LAM concentration and solid media-based CFU count. Specifically, linear regression was performed following log_10_-transformed values of both measures via a mixed-effects modeling approach, shown in Eq. 1, to account for the effects of potential covariates and the presence of potential measurements across multiple study days (that is, Baseline and treatment phases).

1 $${Log}_{10} {CFU}_{i,j}={intercept}_{i}+{Log}_{10} {LAM}_{i,j} \times {slope}_{i}+{\varepsilon }_{j}$$

where: ${Log}_{10} {CFU}_{i,j}$ is the log_10_-transformed CFU count in CFU/mL for the *j*th sample in the *i*th participant; ${Log}_{10} {LAM}_{i,j}$ is the log_10_-transformed LAM concentration in pg/mL for the *j*th sample in the *i*th participant; ${slope}_{i}$ and ${intercept}_{i}$ are the individual participant parameter values for the *i*th participant; and ${\varepsilon }_{j}$ is the individual deviation of the log_10_ ${CFU}_{i,j}$ from the predicted value, based on ${slope}_{i}$ and ${intercept}_{i}$, and is assumed to be N(0, σ^2^).

Individual participant parameter values were assumed to be log-normally distributed, and the interindividual variability (IIV) was modeled using an additive error model. A covariate analysis was conducted to assess the impact of covariates on the variability in sputum LAM concentrations. Covariates included the evaluation of effect of study day (Baseline, Day 7, and Day 13), evaluation of treatment effects, and evaluation of baseline LAM concentration. Assessment of potential covariates was performed using a stepwise forward selection (α = 0.05) and backward elimination (α = 0.01) method. Covariate effects were added to the model in a multiplicative manner via nonlinear (power model) relationships for continuous covariates while categorical variables were evaluated as a proportional shift. Given the expectancy of measurement errors in both the independent and dependent response variables, a simulation-extrapolation (SIMEX) approach was utilized to compensate for assay noise. This approach was applied as described in Bonate [15]. The assay variability, σ_assay_, was specified as 17.8%, the maximum inter-assay %CV observed in the quality control samples from the NexGen EBA cohort.

For all analyses, standard goodness-of-fit (GOF) plots and criteria (that is, r^2^, Akaike information criteria [AIC], and likelihood ratio test) were utilized to evaluate whether the log–log regression was sufficient or if additional model terms needed to be added. The adequacy of the final model was evaluated using a simulation-based, visual predictive check (VPC) method [16, 17]. Monte-Carlo simulations were performed by simulating 1000 datasets identical in structure to the original dataset using NONMEM. Model evaluation was performed throughout the modeling steps via VPC, which provided a graphical model performance assessment.

#### Relationship between sputum LAM concentrations and MGIT assay

As the time-to-event framework has previously been applied for the analysis of MGIT-TTD data with good results [18], a similar approach was utilized for this analysis. Kaplan–Meier (K-M) estimates for the probability of detecting a positive signal in the MGIT assay versus MGIT incubation time stratified by LAM concentration quartiles were assessed for trends and used to guide an optimal and logical model-building strategy. The effect of LAM concentration on the probability of a positive MGIT signal was evaluated using a semi-parametric Cox proportional hazards model. LAM concentration as a predictor of MGIT-TTD was evaluated (α = 0.05) as a continuous variable and as a categorical variable with specified concentration bins. In the former case, linear and log-linear (that is, log_10_ LAM concentration) models linking LAM concentration to MGIT-TTD were evaluated. LAM concentrations below the limit of quantitation were excluded from the assessment of LAM concentration as a continuous variable. It should be noted that the MGIT-TTD endpoint reflects both the total bacterial load and the growth rate of the bacteria. Since LAM concentration is expected to only capture the bacterial load and not the growth rate, evaluations were performed by study day of the sample collection, and treatment was evaluated as an effect on the probability of the event [19, 20].

The assumption of proportional hazards was tested for the Cox proportional hazards model using the correlation between the time to detection and the Schoenfeld residuals. The interaction effect between time and LAM concentration was tested in the model if the Pearson correlation coefficient was statistically significant (α = 0.05) indicating that the proportional hazard assumption was not met. A stepwise forward selection (α = 0.01) methodology was used for the assessment of potential covariate effects. The adequacy of the final model was evaluated using a simulation-based VPC method (500 replicates).

#### Comparison of sputum positivity as assessed by LAM and culture-based methods

The binary endpoint of sputum culture positivity was derived from data on solid media culture, sputum LAM concentration, and MGIT (solid media ± , LAM ± , and MGIT ± , respectively). Analyses based on data from all 4 studies were performed (including data from non-TB participants in the Diagnostic studies). Paired samples were used to make comparisons for the following combinations: solid media ± versus MGIT ± , solid media ± versus LAM ± , MGIT ± versus LAM ± , and solid media plus MGIT-TTD ± versus LAM (that is, “ + ” if either MGIT or solid media culture is positive). Receiver-operator curve (ROC) analysis was performed, including the calculation of the area under the ROC (AUROC). As AUROCs were generated from paired measurements in the same individuals, comparisons between AUROCs were based on the method of DeLong, DeLong, and Clarke-Pearson [21].

#### Software and hardware

Data handling and analyses were conducted using R Version 3.6.1 [22], as implemented via RStudio Server Pro Version 1.3.1056-1 [23], and NONMEM Version 7, Level 3.0 [24]. NONMEM analyses were performed on an Intel cluster with the Linux operating system. KIWI Version 4 [25] was utilized for the mixed-effects modeling approach. The R package *coxph* [26, 27] was used to perform the regression analysis of survival data, while *pROC* [28] was used to compare AUROCs.

## Results

For the quantification of sputum LAM concentrations as a biomarker for bacterial load, 928 LAM concentrations obtained from direct sputum samples and 1199 LAM concentrations obtained from sputum sample sediments were included. For solid media-based sputum culture results, 2323 observation records of CFU counts, and for liquid media-based sputum culture results, 2910 MGIT-TTD values were available for the current analysis. Table 1 shows an overview of all the cohorts and observations included in the current analysis.

### Correlations between assays

Comparisons between LAM concentrations, direct versus sediment: As a preliminary step, LAM concentrations obtained from direct sputum samples and sediment were compared to one another in paired samples. Results from this comparison showed that sputum LAM concentrations from direct and sediment samples were strongly correlated (Pearson Correlation = 0.9), exhibiting a generally linear trend and an even dispersion around the line of unity (Additional file 1: Fig. S1). Given this strong correlation and the fact that previous analytic validation of sputum LAM concentrations was performed using direct sputum samples [9], the hereinafter presented analyses report LAM concentrations obtained from direct sputum samples only.

**Fig. 1:**
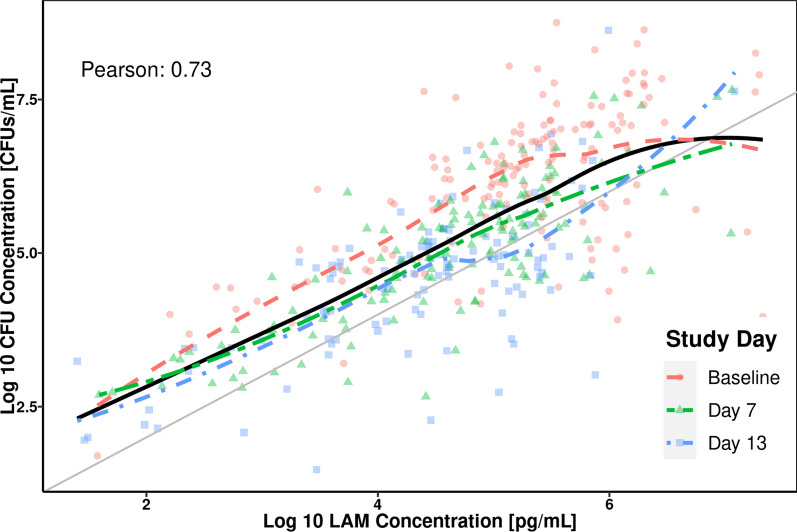
Correlation between log_10_ LAM and log_10_ CFU concentrations in sputum samples from the same visit. Note: Dashed red, green, and blue lines represent spline for Baseline and Days 7 and 13 Visits, respectively. Solid black line represents spline fit to all data. Pearson Correlation shown in figure represents overall correlation. CFU, colony-forming units; LAM, lipoarabinomannan; log_10_, 10 logarithm

Comparisons between sputum LAM concentrations and solid media-based CFU concentrations: impact of treatment duration in the NexGen EBA cohort: Sputum LAM concentrations were compared to paired CFU concentrations from solid media culture samples collected on the same study visit in the NexGen EBA cohort (Fig. 1). It is shown that log_10_ CFU concentrations and log_10_ LAM concentrations are strongly correlated with a generally linear trend across the range of data. However, when stratifying by study visit day, a different relationship between log_10_ CFU and log_10_ LAM at baseline was noted versus Days 7 and 13 (Fig. 1).

**Fig. 2:**
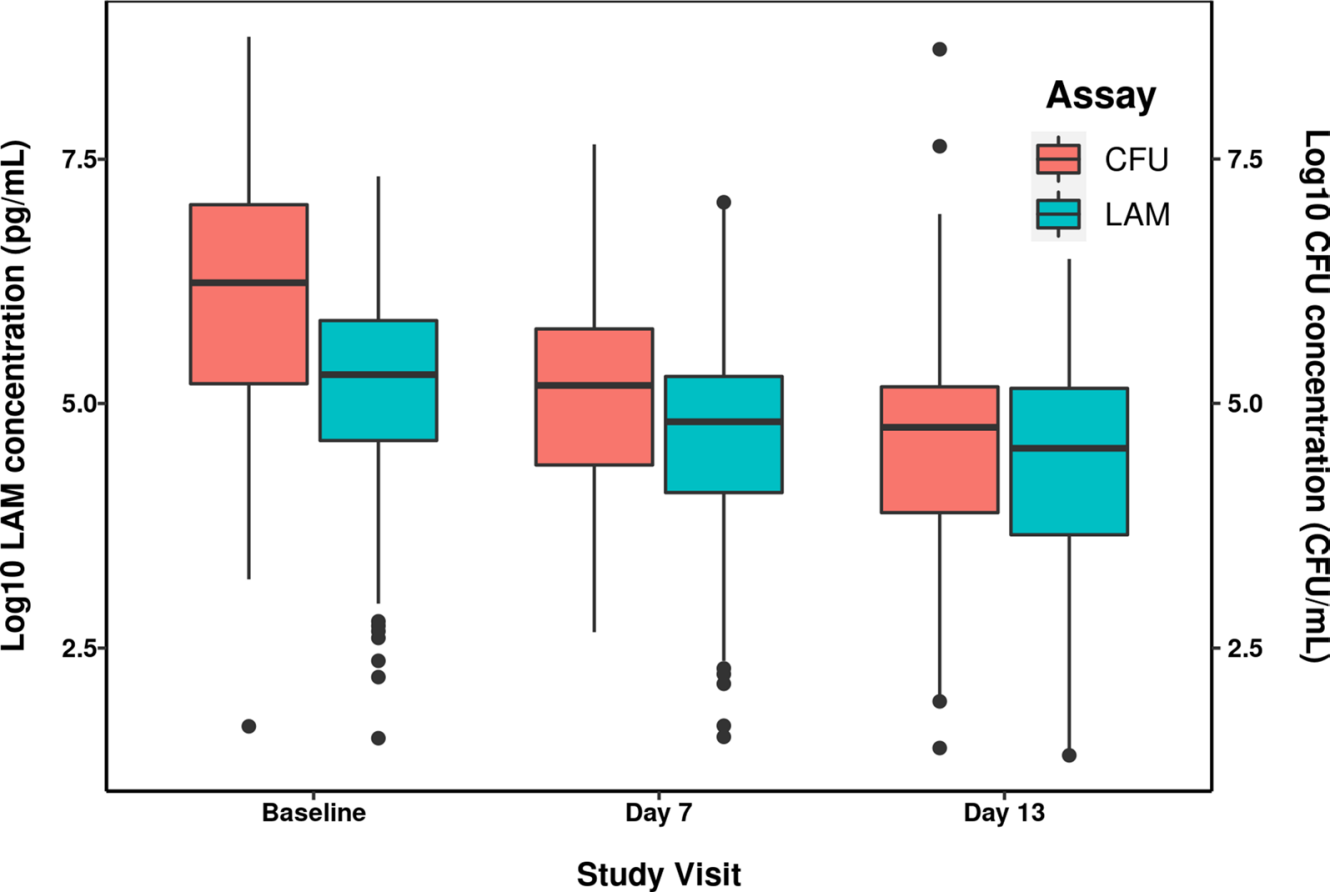
Distribution comparison of log_10_ CFU and LAM concentrations in sputum samples by study visit. Note: Boxes are 25th, 50th, and 75th percentiles, whiskers extend from the hinge to 1.5* inter-quartile range (IQR). Solid black circles are data points outside this range. CFU, colony-forming units; LAM, lipoarabinomannan; log_10_, 10 logarithm

This difference is further seen in Fig. 2 which depicts the distributions of log_10_ LAM concentration and log_10_ CFU concentration in sputum samples on each study visit across all treatment arms in the NexGen EBA cohort. Mean log_10_ CFU concentration was higher than log_10_ LAM concentration on all study days, with the largest difference noted at Baseline and decreasing thereafter. The distribution is wider for log_10_ CFU versus log_10_ LAM concentrations at Baseline, whereas the distribution is similar during treatment (Days 7 and 13). Across all anti-TB regimens, both measures declined during treatment, with a mean decline of approximately 0.8 or 1.5 log_10_ units by Day 13 for LAM and CFU, respectively. Although the magnitude of the changes appears different, the overall trend towards decreasing sputum LAM concentration with treatment mimics that of sputum CFU concentration and is consistent with changes in sputum LAM concentration that have been observed over longer treatment durations with the isoniazid, rifampin, pyrazinamide, and ethambutol (HRZE) regimen (Additional file 1: Fig. S2) [9].

**Fig. 3:**
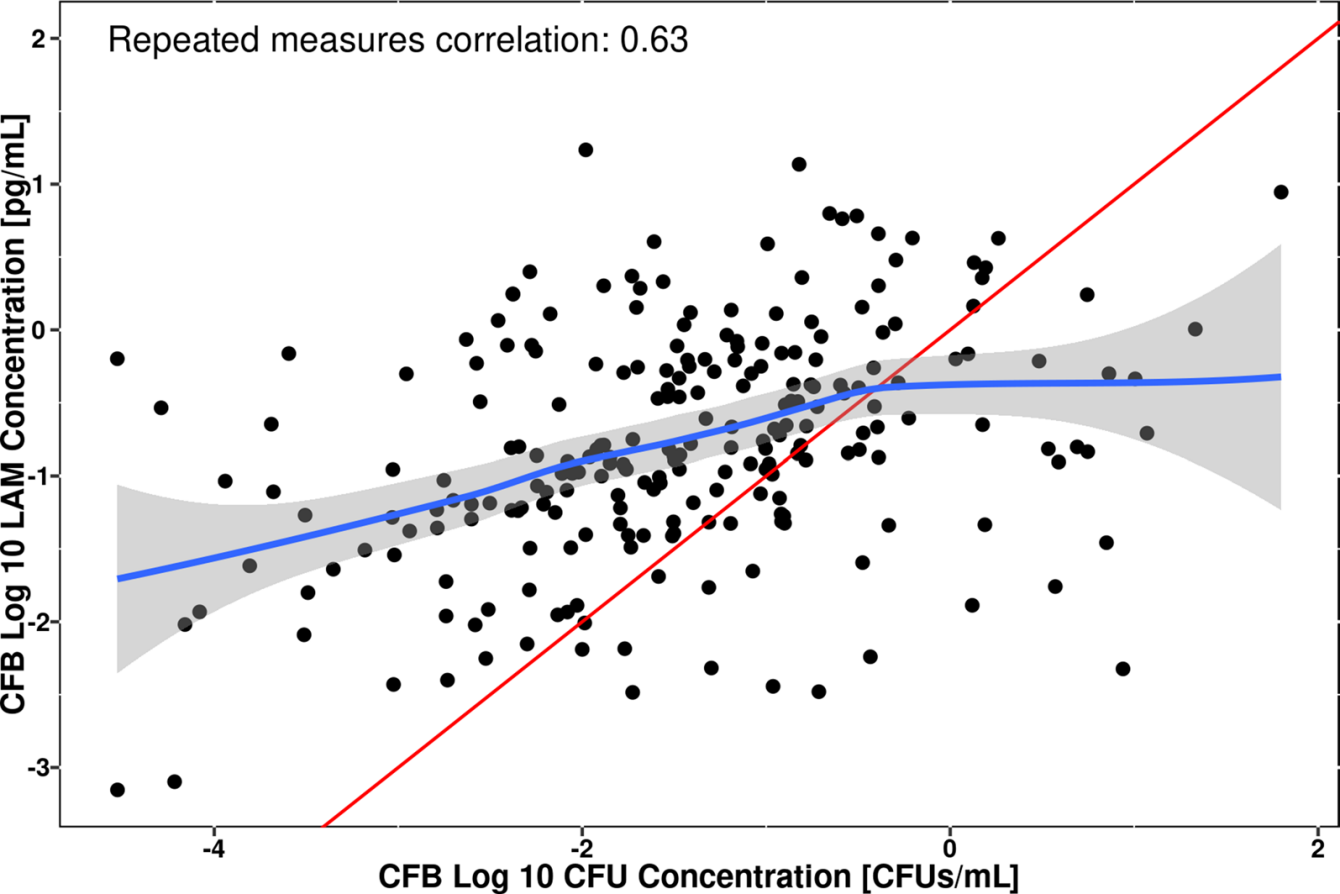
Change from baseline correlation between log_10_ LAM concentrations and log_10_ CFU concentrations in sputum samples. Note: Red solid line depicts line of unity, blue solid line represents a smoothing spline fit to the observed data, gray shaded area depicts the 95% confidence interval of the spline fit. CFB, change from baseline; CFU, colony-forming units; LAM, lipoarabinomannan; log_10_, 10 logarithm

To further compare longitudinal trends for LAM concentration versus CFU concentration in the sputum during treatment, CFB values were derived and compared in Fig. 3. A moderate correlation between CFB log_10_ CFU and CFB log_10_ LAM sediment with a general linear trend is apparent (repeated measure correlation = 0.63). However, as seen in Fig. 3, the deviation from the unity line indicates that there is not a 1:1 relationship in the CFB values for LAM versus CFU concentration and therefore differing dynamic response to treatment with anti-TB regimens. Furthermore, this deviation is more apparent at the lower concentrations when compared to the higher concentration ranges, which is consistent with a larger initial CFB seen for CFU versus LAM concentration in Fig. 2 and the day-dependent relationships observed in Fig. 1.

Model-based analysis of the relationship between sputum LAM concentrations and solid media-based CFU concentration: impact of treatment regimens in the NexGen EBA cohort: The impact of treatment regimens was evaluated by examining whether treatment regimen was a statistically significant covariate in the analysis, as described in the Methods section. Time-matched log_10_ LAM concentrations and log_10_ CFU concentrations from 396 samples were available from 163 participants for this analysis. Application of the initial structural model (Eq. 1) revealed strong correlations between the intercept and slope terms. To adjust for this correlation and enable estimation of the intercept and slope values, the model was adjusted to center the log_10_ LAM concentrations at the median value (4.995 pg/mL). Interindividual variability was estimated for the intercept term with reasonable precision (that is, percent relative standard error [%RSE] < 30%), whereas IIV was not supported for the slope term. Residual variability was described using an additive residual error model.

Univariate forward selection was performed in order to explore the sources of variability in log_10_ LAM concentrations and covariates were tested on the intercept and slope. Consistent with the findings presented in Fig. 1, observation day (Baseline, Days 7 and 13) was selected as a significant covariate during the forward selection process. However, as estimation of different intercepts for all 3 occasions resulted in numerical difficulties, the study day covariate was dichotomized to a shared effect of Days 7 and 13 versus Baseline, which resulted in a more stable model. The different treatment regimens included in the NexGen EBA cohort were explored as potential covariates during the forward selection process but did not result in a significant model improvement. Specifically, the treatment effect was neither statistically significant when estimated independently (that is, separate terms for each of the 8 different regimens) nor when estimated following grouping of treatments together based on their estimated effects and significance thereof. No other covariates were found to be significant during forward selection, and the dichotomous effect of Baseline versus Days 7 and 13 on the intercept term was retained during the backward elimination phase.

The fixed and random parameter estimates for the final mixed-effects model and their %RSE values are shown in Table 2, with corresponding GOF plots provided in Additional file 1: Fig. S3. Overall, the parameter estimates and GOF plots demonstrate that the model fit was reasonable and essentially unbiased, with no significant trends or signs of substantial mis-fit evident in any of the plots. It was considered, however, that both the dependent and independent variables in this analysis represented concentrations derived from laboratory-based measurements with associated assay variability, and therefore a SIMEX approach was utilized to compensate for LAM-ELISA variability on the regression-based estimates (Additional file 1: Fig. S4). The results from the SIMEX analysis (Table 3) show that the model-estimated parameter value and the SIMEX-adjusted parameter value are very close for all parameters in the model, with the ratio of SIMEX to observed values ranging between 0.96 and 1.03. The observed assay variability in the NexGen EBA cohort is larger than previously reported (17.8% versus 5.1%) [9], likely due to the use of multiple lots of manufactured ELISA plates and reagents. Even with this larger variability, the impact of assay noise on the estimated model parameters is minimum.

**Table 2:** Parameter estimates of the mixed-effects model of log_10_ LAM concentration and log_10_ CFU concentration

Parameter	Final parameter estimate		Magnitude of IIV	
	Population mean	%RSE	Final estimate	%RSE
INT: Intercept	5.99	1.26	0.521 SD	22.2
Day 13	− 0.806	11.1	NE	NE
SLP: log_10_ LAM Slope (Centered at Median)	0.763	7.12	NE	NE
Residual Variability (Additive)	0.480 (0.693 SD)	11.8	NA	NA

CFU, colony-forming units; IIV, interindividual variability; LAM, lipoarabinomannan; log_10_, 10 logarithm; NA, not applicable; NE, not estimated; %RSE, relative standard error expressed as a percent; SD, standard deviation

**Table 3:** SIMEX estimates for the mixed-effects model of log_10_ LAM concentration and log_10_ CFU concentration

Type	Intercept	log_10_ LAM Slope (Centered at Median)	Shift for Intercept Day 7 and Day 13	IIV Intercept (Variance)	Residual Variability
Observed (Estimated Mixed Effect Parameter)	5.986	0.763	− 0.806	0.271	0.480
SIMEX Simulation	6.038	0.795	− 0.788	0.264	0.465
Ratio (Observed/ SIMEX)	0.991	0.960	1.022	1.026	1.031

CFU, colony-forming units; IIV, interindividual variability; LAM, lipoarabinomannan; log_10_, 10 logarithm; SIMEX, simulation-extrapolation

**Fig. 4:**
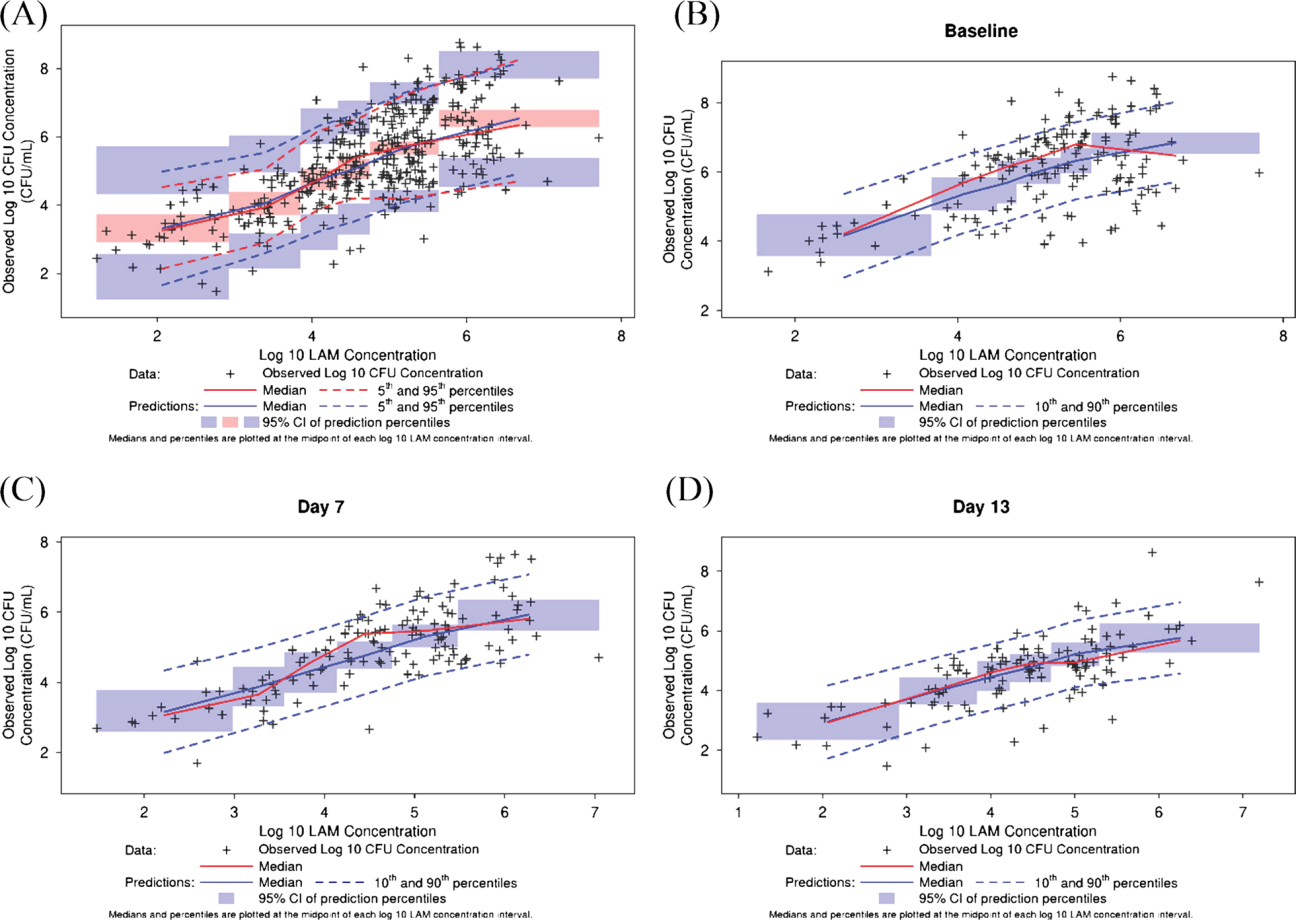
Visual predictive check of the mixed-effects model of log_10_ LAM concentration and log_10_ CFU concentration. CFU, colony-forming units; CI, confidence interval; LAM, lipoarabinomannan; log_10_, 10 logarithm

The predictive performance of the mixed-effects model was evaluated via VPC using the SIMEX-adjusted model parameters. The VPC results shown in Fig. 4 indicate that the linear mixed-effects model for the relationship between log_10_ LAM concentration and log_10_ CFU concentration adequately characterized both the central tendency of the data over the entire concentration range, as well as the extent of variability in the observed data. Figure 4 A depicts the overall VPC while Fig. 4 B–D depict the VPC stratified by Baseline and Days 7 and 13, respectively. Overall, it is shown that there is good agreement between the predicted and the observed log_10_ CFU concentrations. The current analysis did not show any treatment effects, indicating that sputum LAM concentration sufficiently accounted for the treatment-specific effects in the NexGen EBA study.

### Relationship between sputum LAM concentration and MGIT assay

**Fig. 5:**
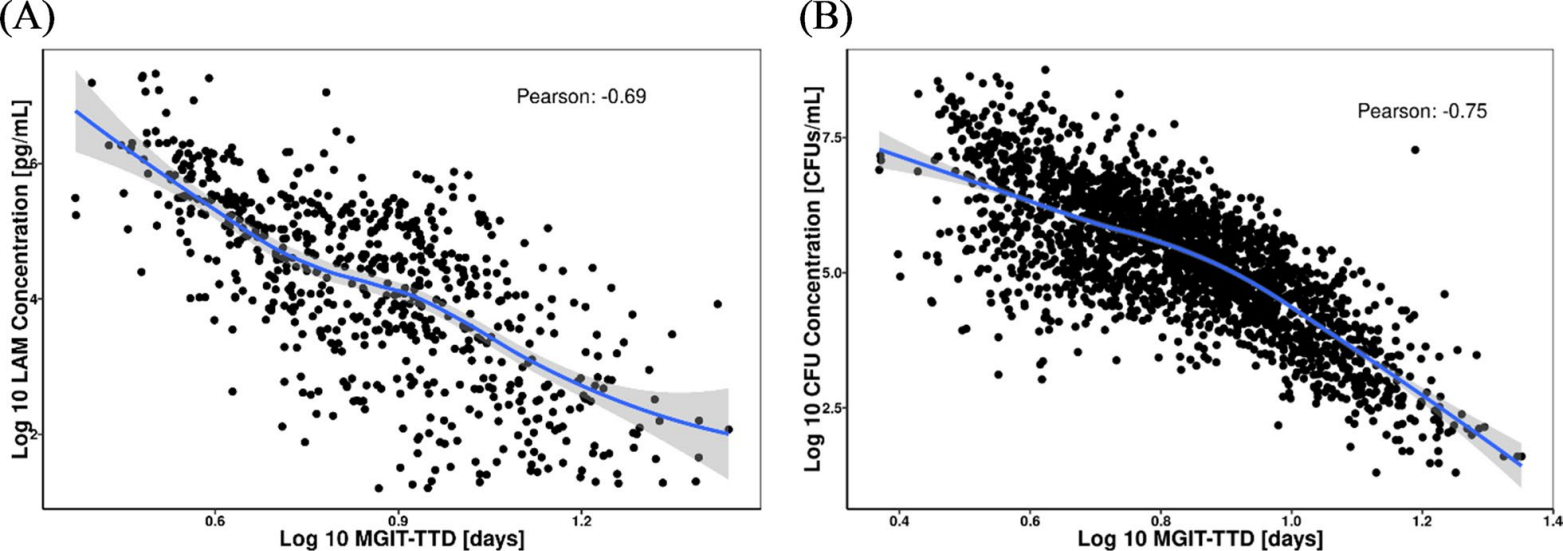
Correlation between paired log_10_ LAM concentration (**A**) and CFU concentration (**B**) versus log_10_ MGIT-TTD. CFU, colony-forming units; LAM, lipoarabinomannan; log_10_, 10 logarithm; MGIT, Mycobacterium Growth Indicator Tube; TTD, time to detection

A total of 853 time-matched LAM concentrations and MGIT-TTD values were available from 476 participants across 3 cohorts (see Fig. 5A). As was performed for LAM concentrations and CFU concentrations, the relationship between these 2 measures was explored graphically and per correlation analysis. Since previous analyses have shown that log_10_ transformation of the MGIT-TTD data improved the robustness of the regression analyses [18], this transformation was applied to evaluate the correlation with log_10_ LAM concentrations. The relationship between log_10_-transformed MGIT-TTD values and LAM concentrations is shown in Fig. 5A, with the corresponding relationship between log_10_-transformed MGIT-TTD values and CFU concentrations shown for comparison (Fig. 5B). These results show that both LAM concentration and CFU concentration exhibit strong negative correlations with MGIT-TTD results. The negative correlation reflects the opposite direction of the measures, in that MGIT-TTD increases with lower bacterial burden. Notably, the correlation between log_10_ LAM concentration (Fig. 5A) and log_10_ MGIT-TTD is nearly identical to that observed for log_10_ CFU versus log_10_ MGIT-TTD (Fig. 5B), albeit with somewhat more variability observed in the former case, especially in the region of lower bacterial burden. Consistent with the relationship between log_10_ CFU and log_10_ LAM concentration, differences in the relationship between log_10_ MGIT-TTD and log_10_ LAM at Baseline versus Days 7 and 13 were apparent (data not shown). This observation, along with the fact that the MGIT-TTD endpoint reflects both the number of bacilli that can grow and their growth rate, prompted the decision to perform the analysis of LAM concentration as a predictor of MGIT-TTD with stratification by study visit.

In the analysis of the relationship between sputum LAM concentration and MGIT-TTD, it was considered that application of the same linear mixed-effects modeling approach utilized to quantify the LAM versus CFU concentration relationship was not appropriate. This is because the result provided from the MGIT assay is not a concentration but rather the incubation duration at which a sample exhibits a positive growth signal or is considered negative (≥ 42 days by convention). Hence, the occurrence of a positive signal in the MGIT assay can be viewed as an “event,” the probability of which is related to the amount and growth rate of viable *Mtb* growing in the assay media. Hence, MGIT-TTD is essentially a “time-to-event” endpoint as often seen in clinical trials, with right censoring occurring at the end of the trial (or in this case, the end of the incubation period). As standard analysis methodologies for time-to-event data (that is, K-M analyses and proportional hazard models) are routinely applied in the analysis of clinical trial data and have previously been applied for the analysis of MGIT-TTD data with good results [18], a similar approach was utilized for this analysis.

**Fig. 6:**
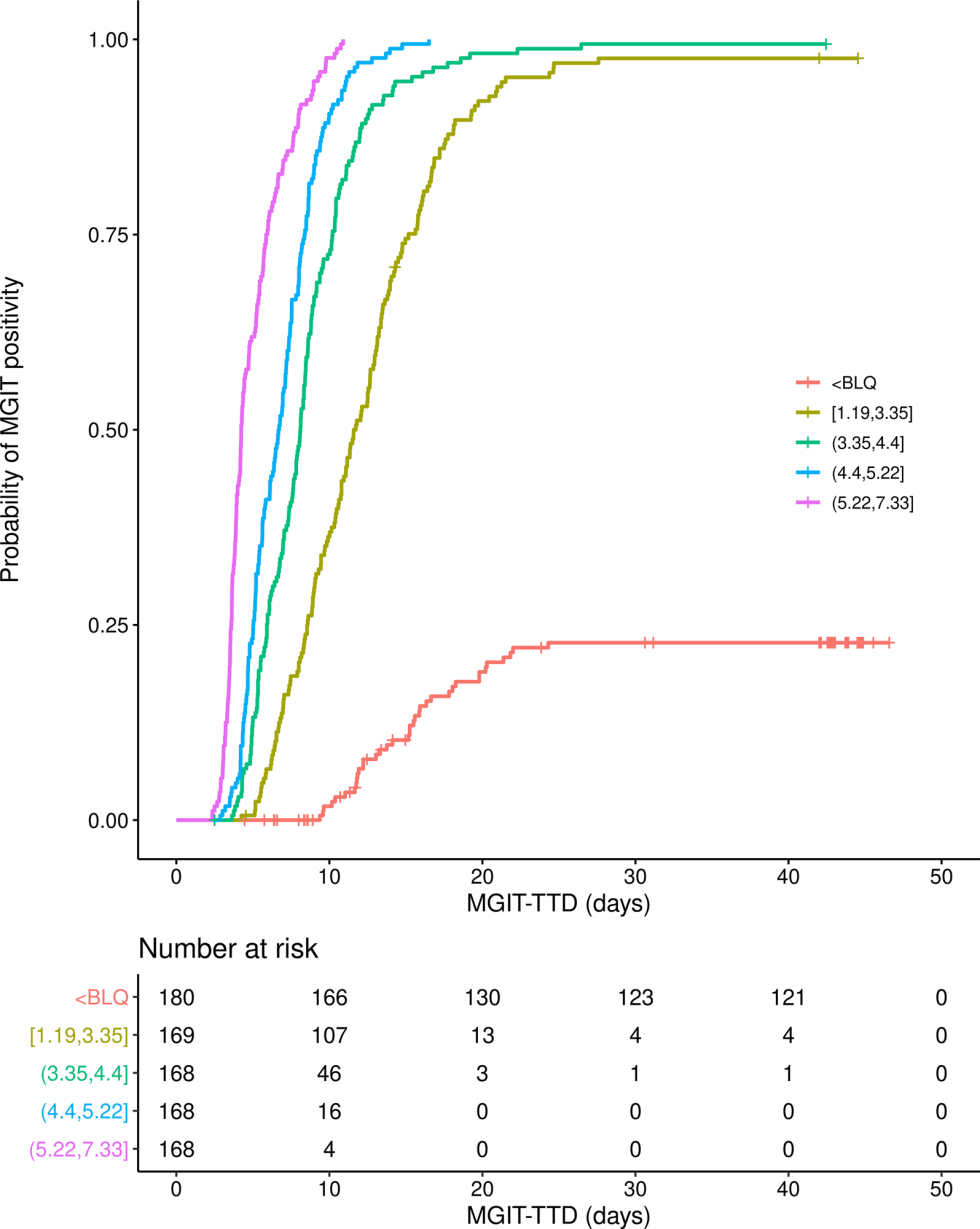
Kaplan–Meier plot of MGIT positivity versus MGIT-TTD, stratified by log_10_ LAM concentration quartiles. Note: [or] indicates respective endpoint is included in the interval and (or) indicates respective endpoint is not included in the interval. BLQ, below the lower limit of quantitation; LAM, lipoarabinomannan; log_10_, 10 logarithm; MGIT, Mycobacterium Growth Indicator Tube; TTD, time to detection

Overall, 702 events (that is, MGIT-TTD < 42 days) occurred and 151 observations had MGIT-TTD ≥ 42 days. Kaplan–Meier curves shown in Fig. 6, displaying the probability of a positive MGIT signal stratified by log_10_ LAM concentration quartiles, indicate that across all studies, regardless of treatment or observation day, increasing sputum LAM concentration is associated with a shorter median MGIT-TTD value. Given the clear relationship seen in the K-M analysis, a semi-parametric (Cox) proportional hazard model was used to evaluate the influence of sputum LAM concentrations on MGIT-TTD.

For model development, the dataset was stratified by nominal observation time (that is, Baseline, Day 3, Day 7, Day 13/14, Day 28, and Day 56). Although separate analyses were performed for each study day, consistent results were obtained and therefore for brevity the model results corresponding to 177 sputum samples obtained from 177 patients on Day 7 only are shown hereafter. Kaplan–Meier curves obtained for Day 7 only (Additional file 1: Fig. S5) are consistent with Fig. 6, with increasing sputum LAM concentrations associated with shorter median MGIT-TTD values. Notably, all 177 observations had an event, that is, MGIT-TTD < 42 days, with only 2 samples having sputum LAM concentrations below the LLOQ.

After incorporating the effect of log_10_ LAM concentration in the Cox model, the influence of the treatment regimens on the hazard of a positive MGIT signal was evaluated. In contrast to the lack of treatment effect observed in the linear mixed-effects analysis of sputum LAM concentration versus sputum CFU concentration, several treatment regimens (in reference to HRZE) exhibited statistically significant coefficients: moxifloxacin, rifampin, pyrazinamide, and ethambutol [MRZE], isoniazid plus pyrazinamide [HZ], isoniazid monotherapy [H], and pyrazinamide monotherapy [Z]. The parameter estimates and corresponding precision of the estimates from the Cox proportional hazard model are provided in Table 4. Overall, the model was consistent with the K-M analysis, whereas increasing LAM concentrations were associated with a higher probability of a positive MGIT signal and therefore a decrease in the median MGIT-TTD value. The hazard ratio for the effect of log_10_ LAM concentration was 3.11 (95% confidence interval [CI] = 2.54, 3.81). Notably, the treatment effect estimates presented in Table 4 are listed in ascending order and roughly correspond to the expected rank order of regimen efficacy (bactericidal activity). This suggests that, while sputum LAM concentration is a clear predictor of MGIT-TTD, in the context of a proportional hazards model it is unable to fully account for treatment-related effects on MGIT-TTD. Lastly, although the correlation between time and the Schoenfeld residuals was significant (*P* < 0.001), indicating that the proportional hazards assumption may be violated, inclusion of an interaction term between log_10_ LAM concentration and time to a positive MGIT signal was not statistically significant and was not retained in the model.

**Table 4:** Parameter estimates and standard errors from the final time to detection of MGIT positivity model

Variable	Coefficient	SE	RSE	*P* value	Hazard Ratio (95% CI)
Coefficient of log_10_ LAM Concentration^a^	1.135	0.1028	9.058	0	3.111 (2.544, 3.806)
Coefficients for Treatment effects					
MRZE (N = 19)	− 1.169	0.2942	25.17	7.10E−05	0.3108 (0.1746, 0.5531)
RZ (N = 20)	− 0.08365	0.2779	332.3	0.7634	0.9198 (0.5334, 1.586)
HRZE (reference) (N = 49)	0	–	–	–	–
M (N = 18)	0.4477	0.2859	63.86	0.1174	1.565 (0.8934, 2.741)
R (N = 19)	0.4543	0.2893	63.69	0.1164	1.575 (0.8934, 2.777)
HZ (N = 17)	0.6332	0.2994	47.29	0.03445	1.884 (1.047, 3.387)
H (N = 17)	0.7169	0.2879	40.15	0.01276	2.048 (1.165, 3.601)
Z (N = 18)	2.034	0.3014	14.82	1.51E−11	7.641 (4.232, 13.79)

^a^Hazard ratio is calculated per unit of log_10_ LAM concentration

CI, confidence interval; H, isoniazid; HRZE, isoniazid, rifampin, pyrazinamide, and ethambutol; HZ, isoniazid and pyrazinamide; LAM, lipoarabinomannan; log_10_, base 10 logarithm; M, moxifloxacin; N, number of records; MGIT, Mycobacterium Growth Indicator Tube; MRZE, moxifloxacin, rifampin, pyrazinamide, and ethambutol; *P*, probability; R, rifampin; RSE, relative standard error; RZ, rifampin and pyrazinamide; SE, standard error; Z, pyrazinamide

### Comparison of sputum positivity as assessed by LAM and culture-based methods

**Fig. 7:**
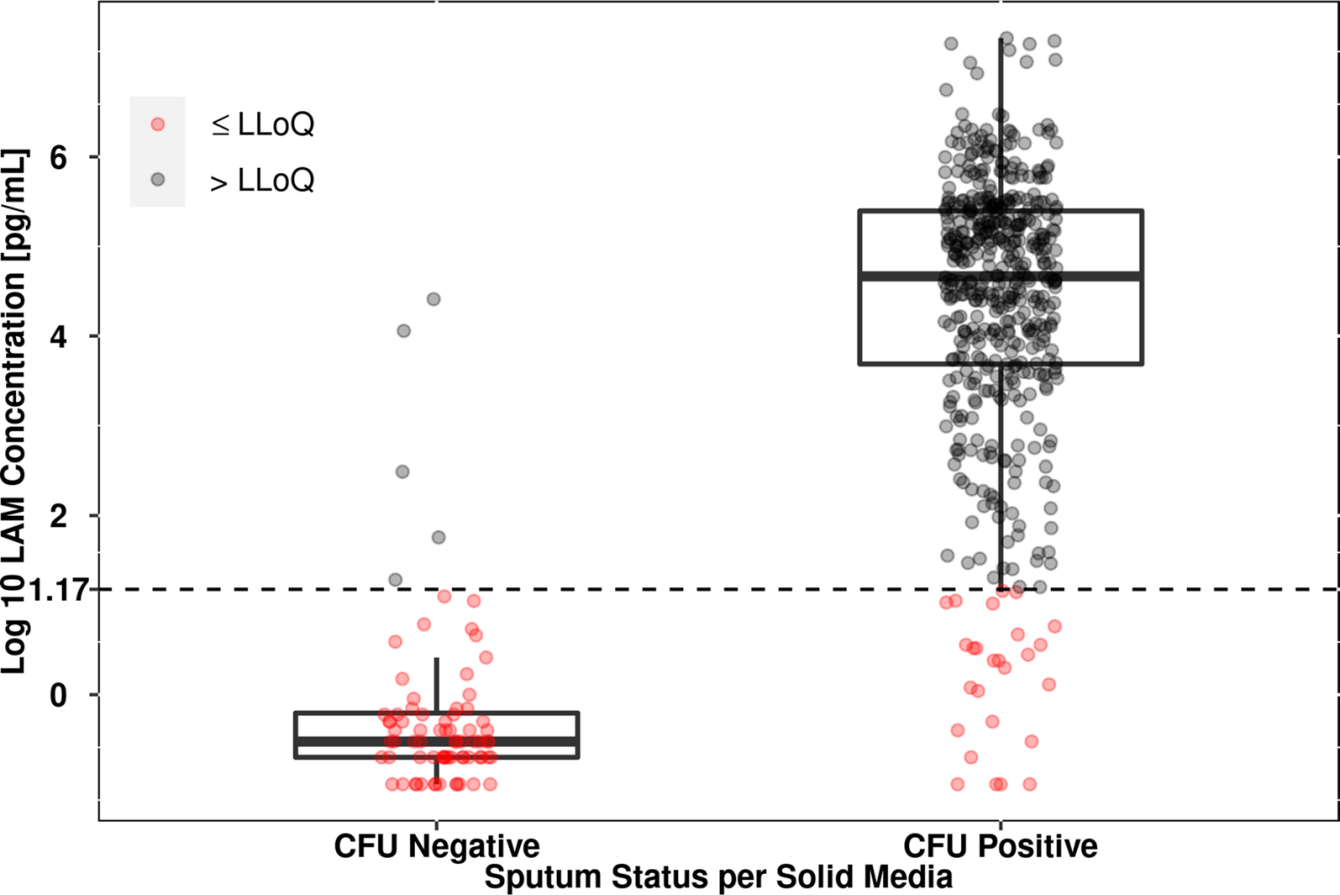
Comparison of log_10_ LAM concentrations by solid media culture status across samples from all cohorts. Note: Dashed line indicates the LLOQ of 1.176 log_10_ pg/mL for sputum LAM concentrations. CFU, colony-forming units; LAM, lipoarabinomannan; LLOQ, lower limit of quantitation; log_10_, base 10 logarithm

**Fig. 8:**
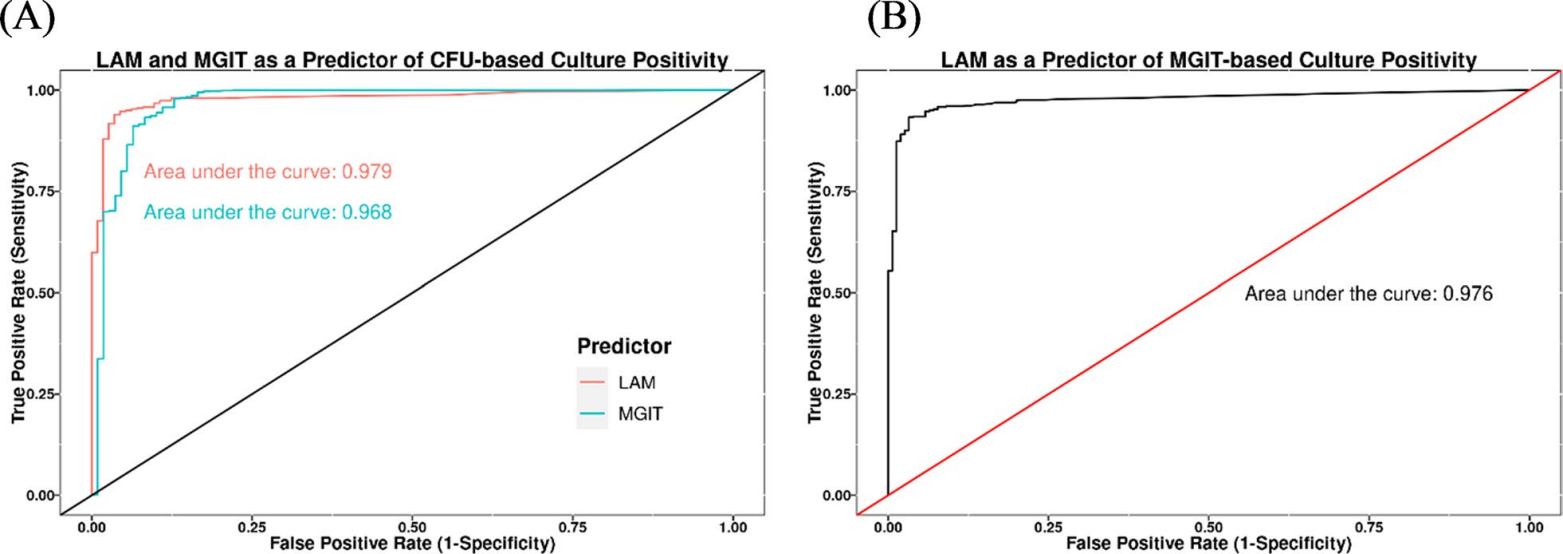
ROCs of LAM and/or MGIT as predictors of CFU-based (**A**) or MGIT-based culture positivity (**B**). CFU, colony-forming units; LAM, lipoarabinomannan; MGIT, Mycobacterium Growth Indicator Tube; ROC, receiver operator curve

The distribution of log_10_ LAM concentrations when stratified by culture positive or negative status as determined by solid media is shown in Fig. 7. As expected, there are clear differences in the distribution of LAM concentrations between the culture positive and negative samples. Furthermore, this is consistent with the ROC analysis of sputum LAM concentration as a predictor of solid media (7H11 or LJ)-based culture positivity (Fig. 8A), which also includes the ROC for MGIT as a predictor of solid media-based culture positivity to aid with interpretation of the ROC results. The ROCs in Fig. 8A are superimposed upon one another, showing that LAM concentration exhibits comparable performance to MGIT as a predictor of solid media-based culture positivity. This is supported by the agreement in the AUROC between both curves, with values of 0.979 and 0.968 for the ROCs with LAM concentration and MGIT as predictors, respectively. Similarly, Fig. 8B shows the ROC for LAM concentration as a predictor of MGIT-based culture positivity (note that an ROC for CFU/mL as a predictor of MGIT-based culture positivity was not provided for reference as the majority of studies did not include quantitative solid media assessments, only categorical or binary assessments). These results show that, as observed for solid media-based culture positivity, LAM concentration is also a good predictor of MGIT-based culture positivity, with an AUROC of 0.976. Table 5 provides the sensitivity and specificity of the various assays as predictors of solid media- or MGIT-based culture positivity. The results in Table 5 show that both the sensitivity and specificity of LAM positivity, defined as values above the LLOQ of 1.176 log_10_ pg/mL, is high for prediction of solid media- or MGIT-based culture positivity and comparable to that seen when solid media-based culture positivity is used as a predictor of MGIT-based culture positivity and vice/versa.

**Table 5:** Quantitative comparison of sputum positivity: sensitivity and specificity across assays

Response	Predictor	Sensitivity	Specificity
CFU Positivity	LAM Positivity	0.944	0.956
	MGIT Positivity	0.995	0.926
MGIT Positivity	CFU Positivity	0.92	0.947
	LAM Positivity	0.993	0.942
CFU or MGIT Positivity	LAM Positivity	0.934	0.942

CFU positivity is defined as the presence of visible growth on solid media, MGIT positivity is defined as a MGIT-TTD value less than 42 days, and LAM positivity is defined as a value above the LLOQ of 1.176 log_10_ pg/mL

CFU, colony-forming units; LAM, lipoarabinomannan; LLOQ, lower limit of quantitation; MGIT, Mycobacterium Growth Indicator Tube; TTD, time to detection

## Discussion

There is an increasingly recognized need for a rapid measure of treatment response to expedite TB drug/regimen development [4]. Such measures fall in the category of PD biomarkers and have a particularly important application in providing rapid evaluation of treatment response during the conduct of early phase clinical trials, thereby enabling meaningful clinical trial adaptations in the context of new anti-TB drug and regimen development. Sputum LAM concentration has been identified as a promising rapid PD biomarker [9], but the available data to support the use of sputum LAM as a PD biomarker in drug development was limited. Here, we analyzed the correlations of sputum LAM and quantitative culture in a multi-arm EBA study and performed exploratory and model-based analyses by combining data from this EBA cohort with 3 other cohorts. Analyses presented herein support the potential of sputum LAM as a PD biomarker during TB treatment.

In the present analysis, it was shown that sputum LAM concentration is a good predictor of both quantitative CFU/mL values as well as binary CFU-based culture positivity. Sputum LAM concentration was shown to be highly correlated with CFU/mL (Fig. 1) with treatment-dependent changes from baseline during short treatment durations. Despite this correlation, different initial changes from baseline between sputum LAM and sputum CFU/mL concentrations were noted in Fig. 3, consistent with a larger initial CFB seen for CFU versus LAM concentration (Fig. 2) and the observed study day-dependent relationships (Fig. 1). This precluded the performance of a Bland–Altman analysis, which is typically performed when assessing the agreement between assays [29]. Rather, a mixed-effects modeling approach was applied, which successfully adjusted for the time-dependence of the sputum LAM and sputum CFU concentration relationship and enabled exploration of additional treatment effects. The model-based analysis showed that the linear mixed-effects model reliably captured the relationship between log_10_ LAM concentration and log_10_ CFU/mL and furthermore, allowed for estimation with good confidence (≤ 22.2%RSE). In this regard, while it is tempting to condense the results of the model-based analysis to the level of a conversion factor that changes sputum LAM concentration into “CFU/mL equivalents,” the model results do not support this simplification. This is because in the power model represented by the log_10_-log_10_ regression, the parameter, α, represents the ratio between CFU count (in CFU/mL) to sputum LAM concentration (in pg/mL) at a LAM concentration of 1 pg/mL. In this regard, it is noted that α would be considered equivalent to the “conversion factor” of 8.06 CFU/mL cited in Kawasaki, et al. [9]. However, such a conversion factor is only valid under the assumption of a generally linear relationship between CFU count and LAM concentration (that is, a β value ≈ 1) per Eq. 1. Since the slope value (equal to β) was estimated at 0.763, this assumption was not met and, thus, the 8.06 CFU/mL conversion factor cited in Kawasaki, et al. is a general approximation only. In other words, rather than using a fixed conversion factor, the model-based approach described in the current analysis is required for conversion of sputum LAM concentration to CFU/mL in order to account for the non-linearity in the CFU/mL versus LAM relationship.

In consideration of the mixed-effects model results, the temporal effect manifested by the covariate of visit day in the NexGen EBA study equates to more CFU/mL per each pg/mL of LAM at Baseline versus at later timepoints (Day 7 and Day 13), or in other words, a higher ratio of LAM to CFU during treatment. Although the basis for this effect is unclear, it may reflect measurement of LAM from both culturable and unculturable (damaged) bacterial cells during treatment. Regardless of the basis for the temporal effect, the current analysis did not show any residual effects of treatment, indicating that sputum LAM concentration sufficiently accounted for the treatment-specific effects in the NexGen EBA study. While this suggests that sputum LAM can be used to estimate the CFU/mL concentrations in the presence of 8 different treatment regimens, these findings are limited by the short treatment duration of 14 days in NexGen EBA. Although sputum LAM concentration has been evaluated over longer treatment durations (that is, in the Kawasaki treatment study, see Table 1), no data are available from studies beyond 14 days that included both sputum LAM concentration and quantitative sputum CFU concentration measurements. Nonetheless, it has been suggested that the relationship between sputum LAM concentration and CFU/mL may hold with longer treatment durations, as inter-study comparisons have shown that the percentage of samples that are positive by sputum LAM or CFU count decreases in a similar fashion over 2 months of treatment with HRZE [14, 30]. This similarity is in line with the ROC analysis shown herein indicating the robust performance of sputum LAM concentration as a predictor of CFU-based culture positivity. Indeed, the predictive performance of sputum LAM concentration indicated by the ROC analysis is on par with that of MGIT-TTD as a predictor of CFU-based culture positivity (Fig. 8), and with comparable sensitivity and specificity at the LAM LLOQ of 15 pg/mL (that is, 1.176 log_10_ pg/mL). Taken together, the results support the premise that sputum LAM concentration can be used to reliably estimate both quantitative sputum CFU concentration and binary CFU-based positivity results.

In addition to the promising findings for solid media-based culture, the present analysis also supports that LAM concentration is representative of results obtained from liquid culture. Sputum LAM concentration is strongly correlated with MGIT-TTD on the log_10_-log_10_ scale, exhibits a high AUROC as a predictor of MGIT assay-based culture positivity and, as noted above, exhibits good concordance with MGIT-TTD as a predictor of CFU-based culture positivity in terms of both ROC analysis and sensitivity and specificity. Further, when viewed in the framework of a time-to-event analysis whereby each positive signal in the MGIT assay is treated as an “event” occurring at the time of the corresponding TTD value, sputum LAM concentration is clearly predictive of MGIT-TTD, with marked differentiation in the K-M plots between each log_10_ LAM quartile and those measurements below the lower limit of quantitation (Fig. 6 and Additional file 1: Fig. S5). This was supported by the results of the Cox proportional hazard model with a hazard ratio for the effect of log_10_ LAM on time to MGIT positivity of 3.11 (95% CI = 2.54, 3.81), indicating that with increasing log_10_ LAM concentration, the probability of MGIT positivity increases and hence time to MGIT positivity significantly decreases.

Although the above results are indicative of a definite relationship between LAM concentration and MGIT-TTD, deeper consideration of the time-to-event analysis presents a more complex picture. Despite the fact that the majority of sputum samples with LAM concentrations below the LLOQ were negative via MGIT assay (that is, right censored at 42 days), approximately 20% of the samples with LAM concentrations less than the LLOQ were detected as MGIT positive, representing a greater false negative rate for predicting MGIT-based culture status as compared to CFU-based culture status (Fig. 7). This suggests that at the current LLOQ, sputum LAM concentration, similar to CFU count, may not be as sensitive as the MGIT-TTD assay in detecting very low numbers of viable *Mtb* in sputum samples. Additionally, treatment-related differences in the time to a positive MGIT signal were not fully accounted for by simply including log_10_ LAM concentration as a predictor alone in the Cox proportional hazard model. It was shown that the inclusion of a treatment effect was warranted, in contrast to the lack of residual effect observed in the case of the linear mixed-effects model linking sputum LAM and sputum CFU concentrations. Given that the latter model results were obtained from the same study cohort as the MGIT samples evaluated in the time-to-event analysis (that is, NexGen EBA), the observation of residual treatment-related effects after inclusion of sputum LAM as a covariate was unexpected. In this regard it is noted that the MGIT-TTD endpoint reflects both the number of culturable *Mtb* bacilli in the sample and their growth rate, as opposed to both sputum LAM concentration and CFU count which are related to only the bacterial load. The additional dependence on growth rate for MGIT-TTD suggests that the treatment effect parameters identified in the time-to-event analysis could reflect the residual effects of the various treatment regimens on growth rate of the culturable *Mtb* population within the MGIT incubations. Although speculative, treatment-induced selection of *Mtb* populations with different cultivability and growth rates in the MGIT assay could explain both the need for treatment effect terms in the time-to-event model as well as the observation of an incubation duration-dependent trend in the Schoenfeld residuals. In the former case, while sputum LAM concentration may be a sufficient predictor of the total number of bacteria, a treatment-specific effect would still be required to account for differences in *Mtb* growth rate in the MGIT incubation. In the latter case, although an interaction term between log_10_ LAM concentration and time to a positive MGIT signal was not statistically significant in the Cox model, it is reasonable to assume that different *Mtb* populations may vary in their incubation duration-dependent probabilities of a positive MGIT signal (that is, exhibit different baseline hazard functions). Therefore, it is possible that as more data become available, the interplay between incubation duration, treatment effect, and LAM concentration suggested by the current analysis may be more clearly elucidated through a parametric time-to-event model analysis that can better account for these complexities. Regardless, based on the current analysis, while sputum LAM concentration is a clear predictor of MGIT-TTD, it does not fully explain the treatment-related differences in MGIT-TTD noted in the NexGen EBA study.

In consideration of the totality of the analyses presented herein, it is important to emphasize that, at present, sputum LAM concentration is not being proposed as a replacement for culture-based methods for assessing response to anti-TB treatment. Rather, it is intended as a PD biomarker to supplement culture results by providing rapid read-outs of treatment effect to inform clinical trial decision making. This context of use is based on the definition of PD/response biomarker per the biomarkers, endpoints, and other tools (BEST) glossary, namely as “*A biomarker used to show that a biological response has occurred in an individual who has been exposed to a medical product or an environmental agent”* [31]. In the drug development setting, a PD/response biomarker may be used to demonstrate target engagement, provide proof-of-concept that a drug may exert a beneficial pharmacologic effect, and guide dose–response studies.

As with any set of post-hoc analyses, there are certain limitations that apply and should be highlighted. Specifically, given the relatively small number of studies to date that have evaluated sputum LAM as a PD biomarker, we opted to pool together multiple cohorts that were designed with different purposes (diagnostic and treatment). While this pooling serves to leverage all available data, it does introduce study-level differences as another variable in the overall interpretation of the results. Additionally, the treatment duration in the included studies is relatively short: 14 days in the NexGen EBA cohort, and with a maximum of 56-day treatment in the pooled dataset. Because of this limited treatment duration, the extrapolation of findings beyond this treatment duration should be done cautiously, and as such we have focused our work on assessing the utility of sputum LAM in early anti-TB drug and regimen development where treatment durations are 56 days or less.

## Conclusions

The summary of evidence presented herein clearly demonstrates that sputum LAM concentration is correlated with bacterial burden in the sputum as measured by both solid media and liquid media culture and reflects changes in bacterial burden in response to pharmacological effect. The rapid availability of sputum LAM changes in response to treatment enables its use in guiding timely clinical trial decision making and, furthermore, may enable adaptive trial designs of anti-TB drugs and regimens. Additionally, since LAM concentrations can be measured in the same sputum samples collected for culture-based assessments, any decisions made on the basis of sputum LAM concentrations can later be verified when culture results become available from the same samples. Therefore, as sputum LAM concentration has significant promise as a valuable tool in the anti-TB drug and regimen development, it is our recommendation that it be included in ongoing [32] and future clinical studies to provide more data and hence better inform its overall context of use.

## Supplementary Information

**Additional file 1.  **Supplementary Results.

## Data Availability

All data generated or analyzed during this study are included in this published article and its additional information files.

## Abbreviations

AFB: Acid-fast bacilli
AIC: Akaike information criteria
AUROC: Area under the ROC
BEST: Biomarkers, endpoints, and other tools
CFB: Change from baseline
CFU: Colony-forming unit
CI: Confidence interval
FIND: Foundation for Innovative New Diagnostics
GOF: Goodness-of-fit
H: Isoniazid monotherapy
HRZE: Isoniazid, rifampin, pyrazinamide, and ethambutol
HZ: Isoniazid plus pyrazinamide
IIV: Interindividual variability
K-M: Kaplan–Meier
LAM: Lipoarabinomannan
LAM-ELISA: Immunoassay developed by Otsuka Pharmaceutical Co*, Ltd.
LJ: Löwenstein–Jensen
LLOD: Lower limit of detection
LLOQ: Lower limit of quantitation
MGIT: Mycobacteria Growth Incubator Tube
MGIT-TTD: MGIT assay-based time to detection
MRZE: Moxifloxacin, rifampin, pyrazinamide, and ethambutol
*Mtb*: *Mycobacterium tuberculosis*
PD: Pharmacodynamics
ROC: Receiver-operator curve
%RSE: Percent relative standard error
SIMEX: Simulation-extrapolation
TB: Tuberculosis
TTD: Time-to-detection
ULOQ: Upper limit of quantitation
VPC: Visual predictive check
Z: Pyrazinamide monotherapy
