# Supplementary material for: Sputum lipoarabinomannan (LAM) as a biomarker to determine sputum mycobacterial load: exploratory and model-based analyses of integrated data from four cohorts
Source: BMC Infect Dis. 2022 Apr 2;22:327. doi: 10.1186/s12879-022-07308-3 (PMC8976459; doi:10.1186/s12879-022-07308-3)
Supplement: Supplementary file 1 — Additional file 1. Supplementary Results. [file 12879_2022_7308_MOESM1_ESM.docx]

Supporting information


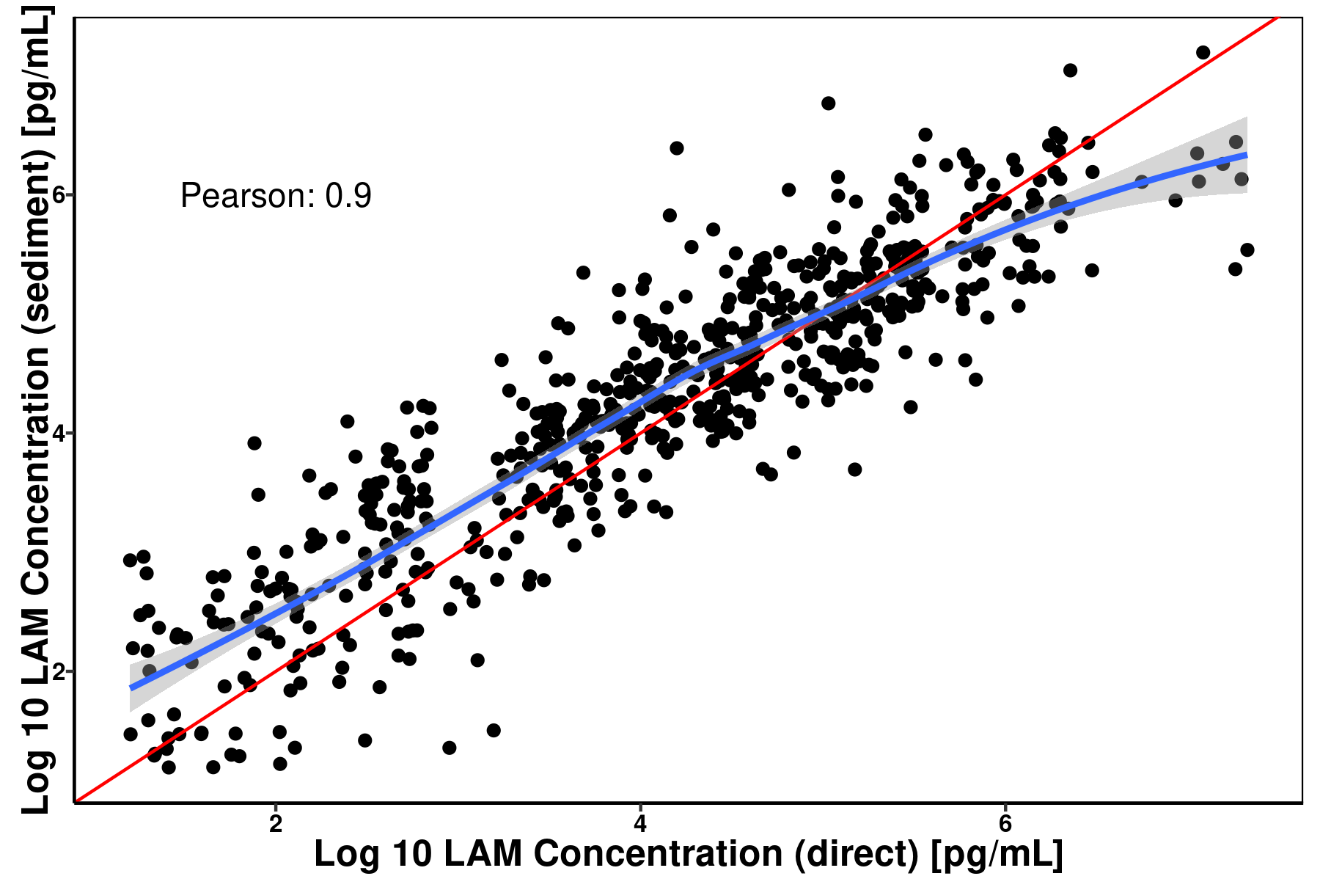


S1 Figure Correlation between paired log_10_ LAM concentrations from sputum sediment and direct sputum samples. Note: Red solid line represents the line of unity; blue solid line represents a fitted spline through the observed data; gray shaded region around spline represents 90% confidence interval, and black solid circles are observed LAM concentrations. LAM, lipoarabinomannan; log_10_, 10 logarithm.


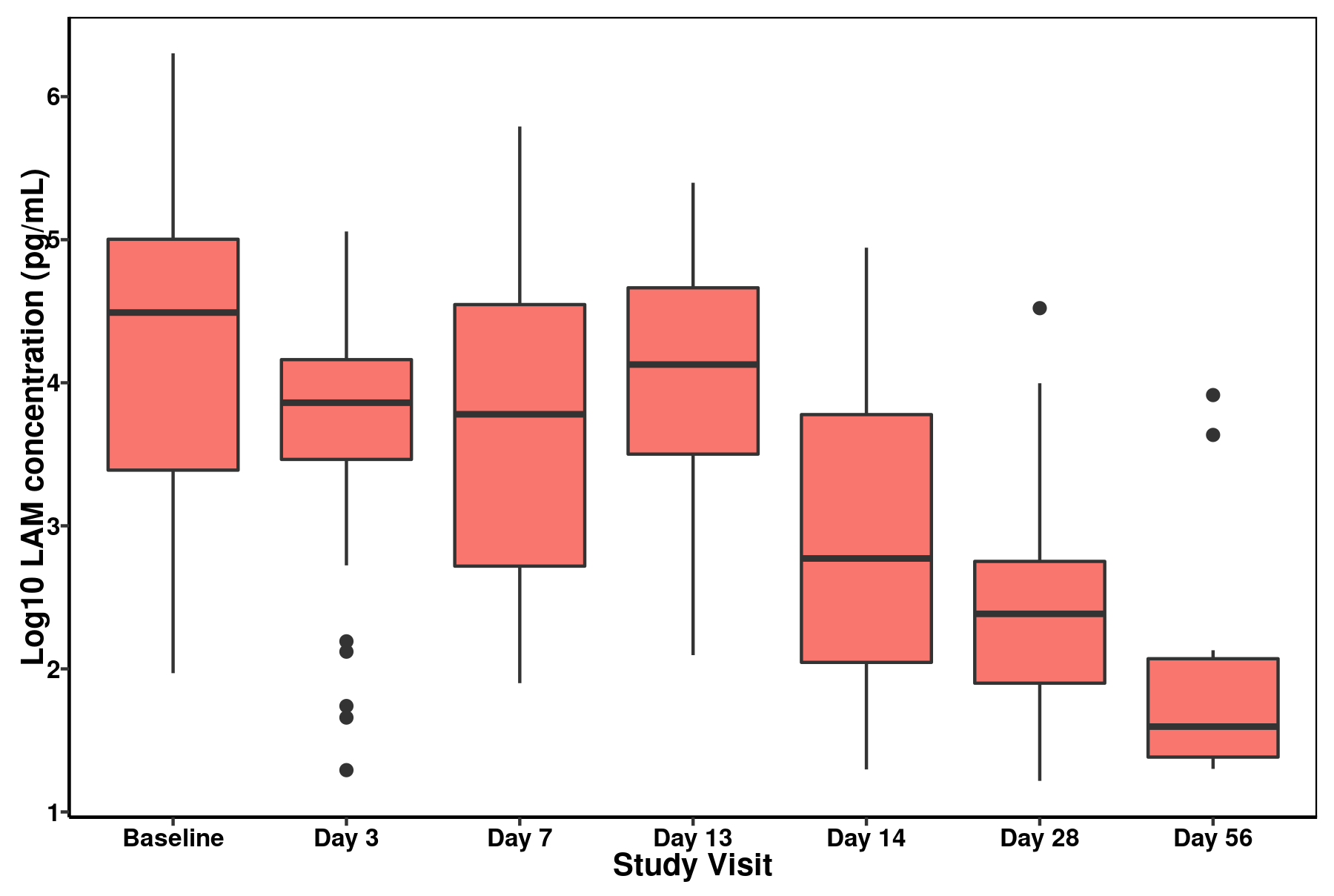


S2 Figure Distribution comparison of log_10_ LAM concentrations in sputum samples across study visits for HRZE regimen. Note: Kawasaki M, Echiverri C, Raymond L, Cadena E, Reside E, Tarcela Gler M, et al. Lipoarabinomannan in sputum to detect bacterial load and treatment response in patients with pulmonary tuberculosis: analytic validation and evaluation in two cohorts. PLoS Med. 2019;16(4):e1002780. HRZE, isoniazid, rifampin, pyrazinamide, and ethambutol ; LAM, lipoarabinomannan; log_10_, 10 logarithm.


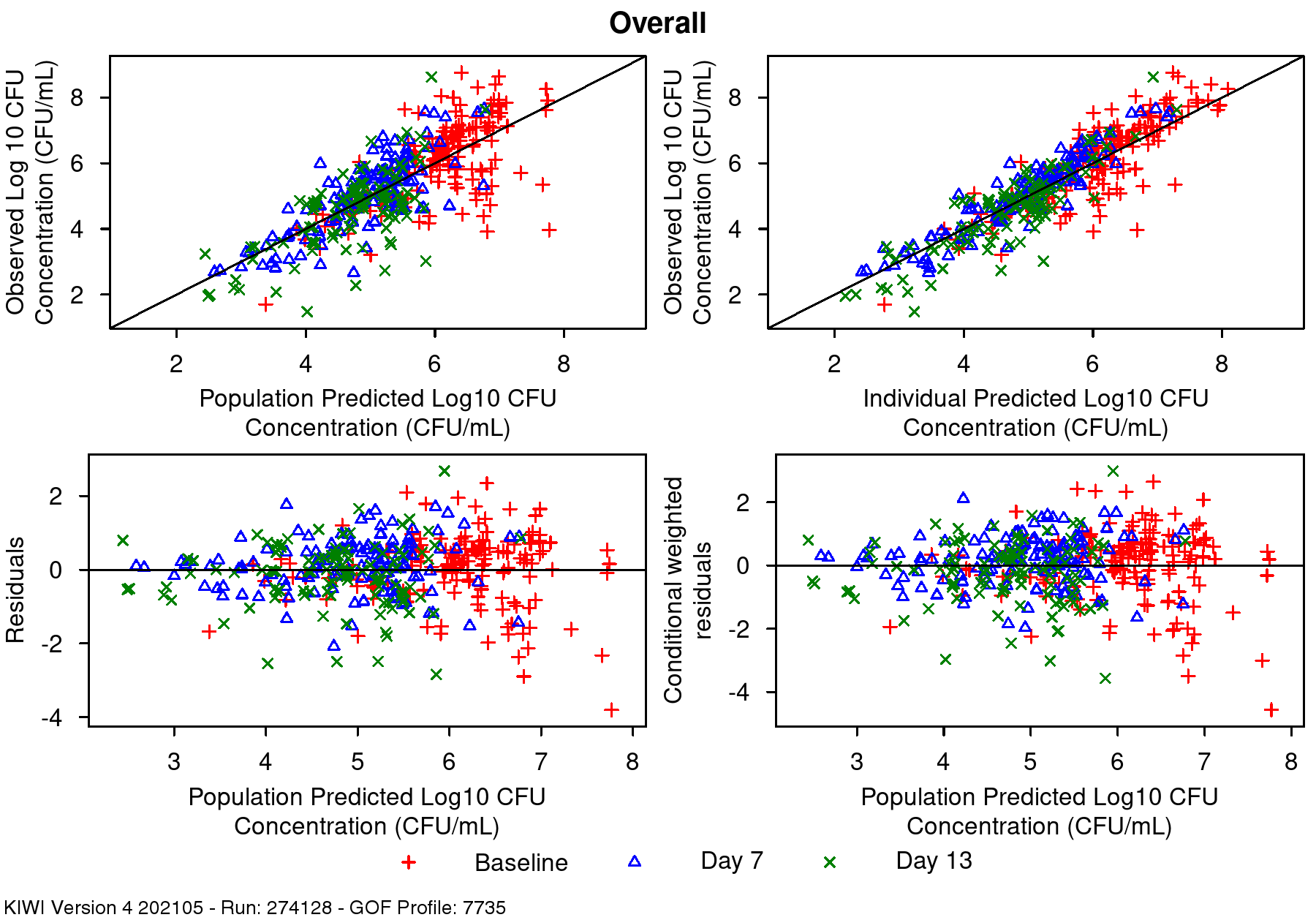


S3 Figure Goodness-of-fit plots for the final mixed-effects model. CFU, colony-forming units; log_10_, 10 logarithm.

(A) (B)


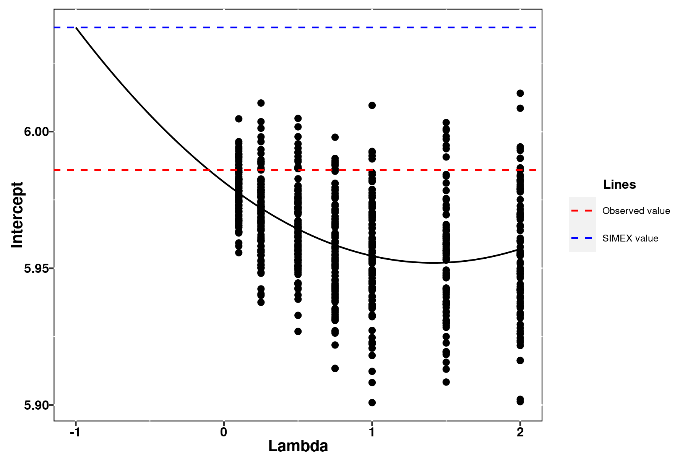

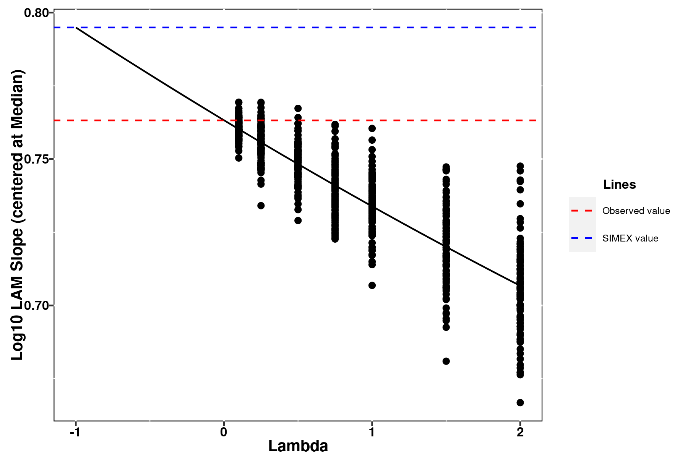
 (C) (D)


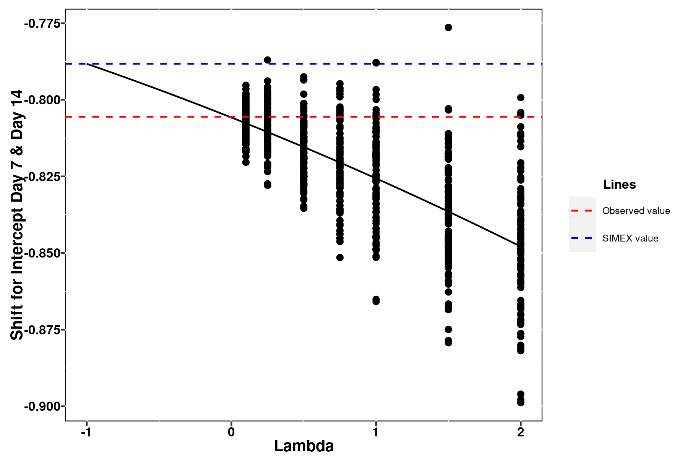

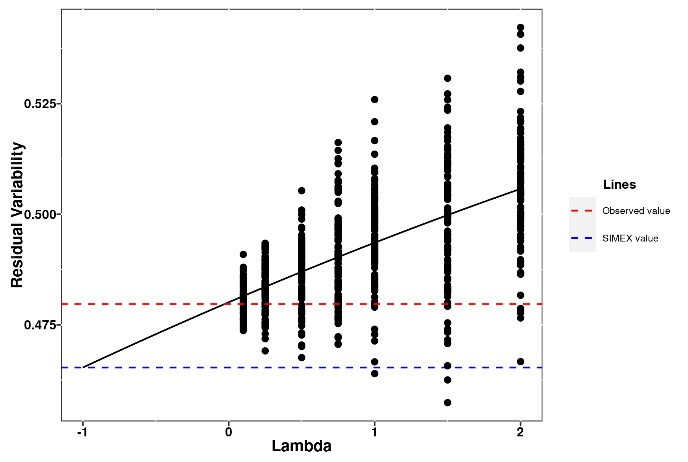
(E)


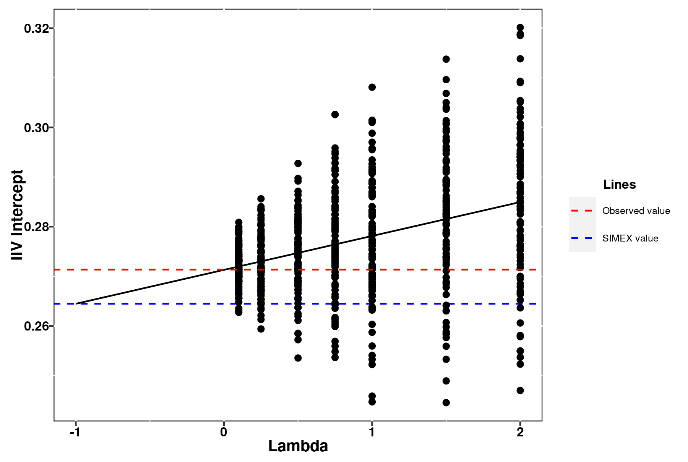


S4 Figure SIMEX parameter estimates as a function of λ. Note: Red and blue dashed lines represent the observed and SIMEX values, respectively; black filled circles and black solid line represent SIMEX parameter estimates and quadratic regression model as a function of λ, respectively. IIV, interindividual variability; LAM, lipoarabinomannan; log_10_, 10 logarithm; SIMEX, simulation-extrapolation.


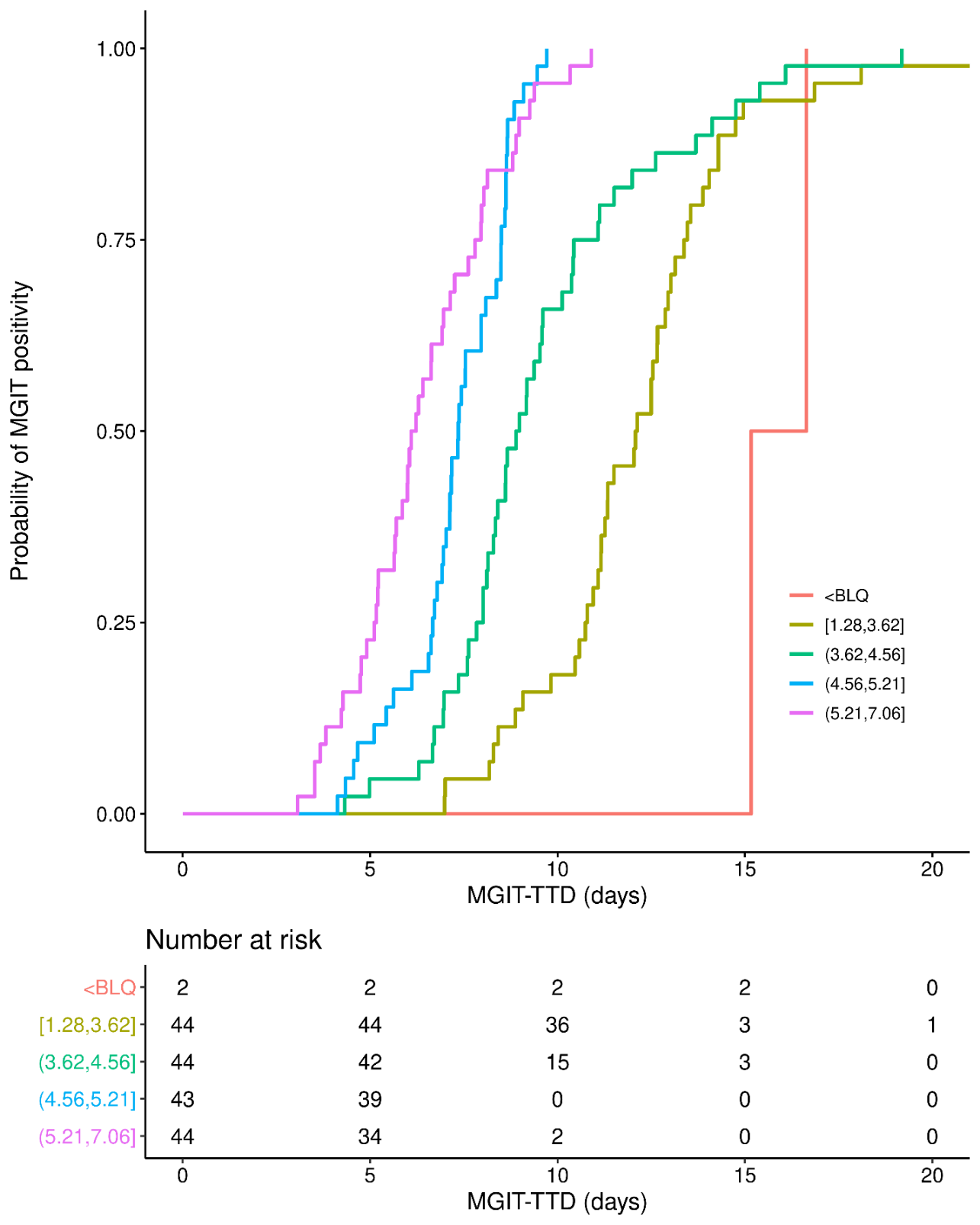


S5 Figure Kaplan-Meier plot of MGIT positivity versus MGIT-TTD, stratified by log_10_ LAM quartiles on day 7. Note: [ or ] indicates respective endpoint is included in the interval and ( or ) indicates respective endpoint is not included in the interval. BLQ, below the lower limit of quantitation; LAM, lipoarabinomannan; log_10_, 10 logarithm; MGIT, Mycobacterium Growth Indicator Tube; TTD, time to detection.
